# Disseminated Gestational Trophoblastic Disease and Diffuse Alveolar Hemorrhage Treated With Extracorporeal Membrane Oxygenation

**DOI:** 10.7759/cureus.8064

**Published:** 2020-05-11

**Authors:** Tim Montrief, Kasha Bornstein, April Grant, Jeffrey M Scott, Ali Ghodsizad

**Affiliations:** 1 Critical Care Medicine, University of Pittsburgh Medical Center, Pittsburgh, USA; 2 Critical Care, University of Miami, Miami, USA; 3 Division of Trauma and Surgical Critical Care, Grady Memorial Hospital, Atlanta, USA; 4 Cardiothoracic and Transplant Critical Care, Miami Transplant Institute, Miami, USA; 5 Division of Heart and Lung Transplant and Mechanical Circulatory Support, University of Miami, Miami, USA

**Keywords:** ecmo, gestational trophoblastic disease, diffuse alveolar hemorrhage, post-partum

## Abstract

Late-stage gestational trophoblastic disease (GTD) bears poor prognosis including acute respiratory distress syndrome (ARDS), multiorgan failure, and death. There are currently no reports of extracorporeal membrane oxygenation (ECMO) therapy for respiratory failure due to disseminated GTD in post-partum patients.

We present a case of newly diagnosed disseminated GTD progressing to ARDS secondary to diffuse alveolar hemorrhage (DAH) for which veno-venous ECMO was successfully implemented.

ECMO is an accepted modality for ARDS refractory to medical therapy. Controversy persists regarding post-partum patient selection as contraindications to ECMO include known poor prognosis. Our case herein suggests that ECMO is an acceptable treatment modality for patients with acute respiratory failure secondary to disseminated GTD. The indications and contraindications for ECMO warrant further discussion and research for post-partum patients.

## Introduction

Treatment of acute respiratory distress syndrome (ARDS) secondary to diffuse alveolar hemorrhage (DAH) in obstetric patients is challenging due to the high mortality risk for both the mother and the fetus. In cases of refractory ARDS unresponsive to conventional measures such as protective ventilation and proning, extracorporeal membrane oxygenation (ECMO) can be a life-saving modality. While the use of ECMO has increased dramatically in the last two decades, the clinical management of these cases remains extremely challenging. We describe one case of ECMO use in a post-partum woman with disseminated gestational trophoblastic disease (GTD).

## Case presentation

**Figure 1:**
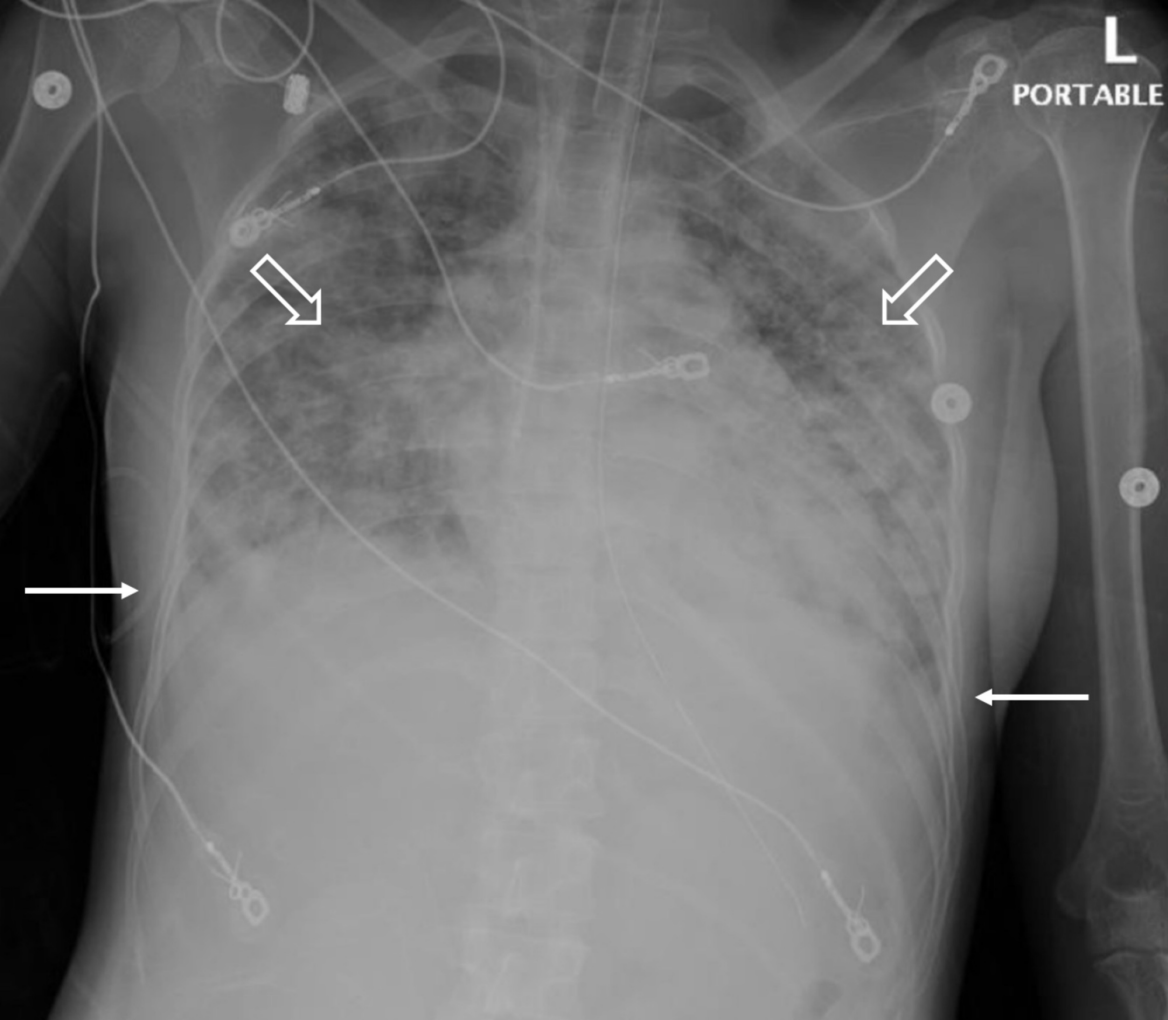
Chest X-ray on hospital day one showing diffuse pulmonary edema (blank arrows), and bilateral airspace disease with bilateral pleural effusions (solid arrows).

Six weeks following an uncomplicated delivery via low-transverse C-section for arrest of labor (placenta not sent to pathology), a 23-year-old G3P2011 woman presented to the ED with pleuritic chest pain and severe shortness of breath for two days. The patient’s prenatal course was unremarkable. Following delivery, she was discharged home on post-operative day three with a hemoglobin (Hgb) of 10.8 g/dL and a hematocrit (Hct) of 32.4%. Her post-partum history was notable for endometritis, excessive vaginal bleeding throughout the post-partum period with evidence of increased endometrial blood flow, as well as an endometrium measuring 1.5 cm via transvaginal ultrasound. She required hospitalization for antibiotics and transfusion of two units of packed red blood cells (PRBCs) two weeks prior, but did not have any curettage performed at that time. Upon initial evaluation, she was afebrile (37.0° C), tachycardic (145 beats per minute), hypertensive [blood pressure (BP) 156/82 mmHg], and in severe respiratory distress with a respiratory rate (RR) of 30 breaths per minute, oxygen saturation of 99% while breathing 100% oxygen via nonrebreather mask. A chest X-ray showed diffuse pulmonary edema and bilateral airspace disease with bilateral pleural effusions (Figure 1).

Initial arterial blood gas analysis showed severe hypoxemia, metabolic acidosis, and concomitant respiratory alkalosis (pH 7.31, pO2 197, pCO2 18, base excess −8, HCO3 18 on 100% O2 via bag-valve-mask). Labs demonstrated a Hgb of 4.9 g/dL and Hct of 15.6% with gross intraperitoneal blood found on sonographic exam, leukocytosis (white blood cell count 16,700 with 82% neutrophils), elevated d-dimer (>5,000 ng/mL), normal fibrinogen, and transaminitis with aspartate aminotransferase/alanine aminotransferase/alkaline phosphatase of 363/356/274 units/L, respectively. Additionally, the patient was found to have a serum beta-human chorionic gonadotropin (hCG) of 2,800 mIU/mL, a creatinine of 1.10 mg/dL, and a lipase of 453 units/L. The patient was transfused with three units of PRBCs, and subsequently intubated with a tidal volume (TV) of 6 mL/kg ideal body weight, a respiratory rate of 16, and a positive end-expiratory pressure (PEEP) of 5 cm H2O and an inspired oxygen fraction of 60%.

**Figure 2:**
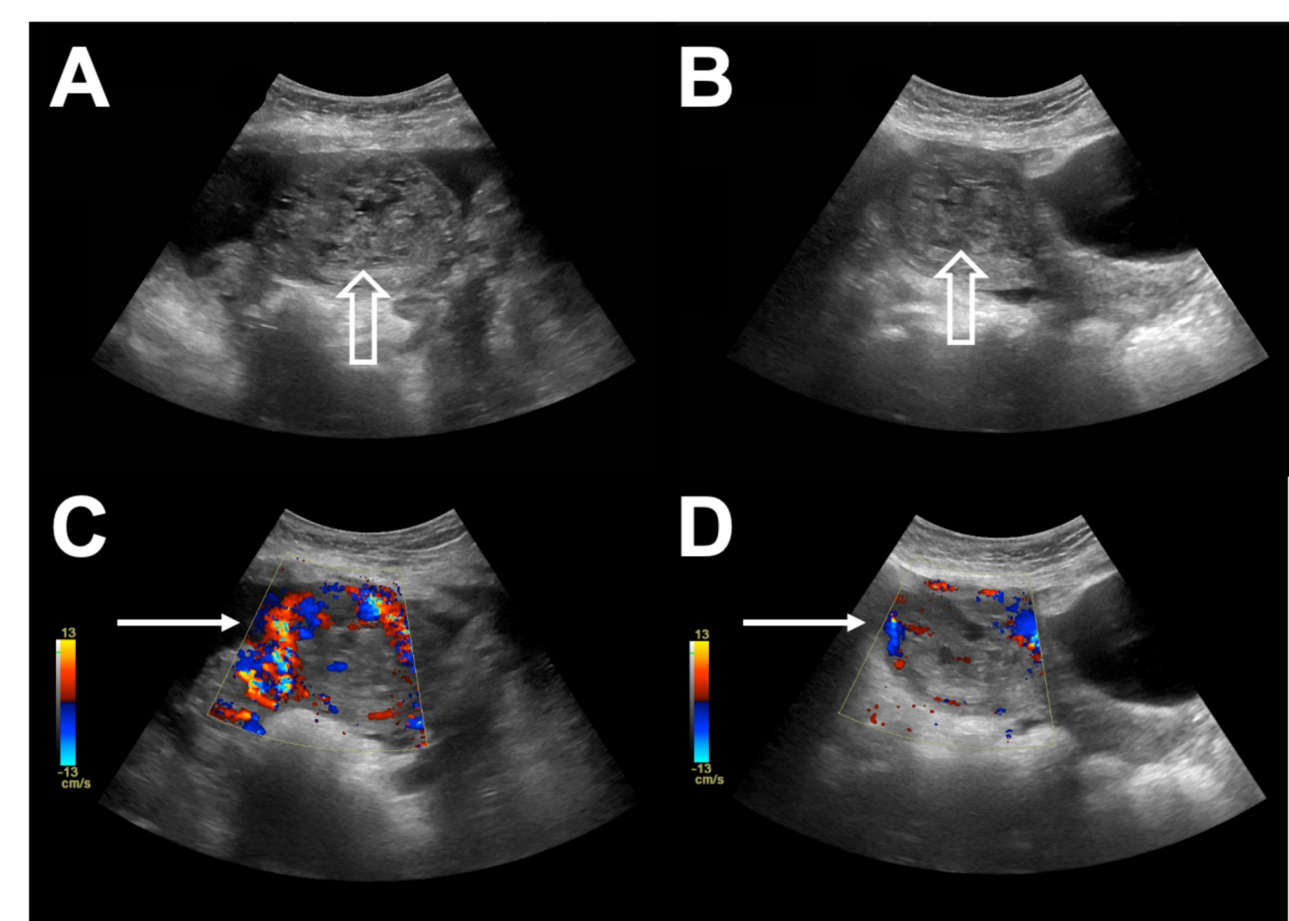
A and B. Initial transabdominal ultrasound, Transverse view (A and C) and sagittal view (B and D) revealing a hypervascular uterus (white arrows) with internal echogenic material (blank arrows).

Initial transabdominal ultrasound revealed a hypervascular uterus with internal echogenic material, as well as an endometrial lining measuring 3.7 cm, suspicious for GTD (Figure 2).

A CT angiogram with contrast of the chest, abdomen, and pelvis showed free fluid in the abdomen, multiple lesions within the liver, spleen, bilateral pleural effusions, and bilateral thromboses in the gonadal veins, raising concern for septic emboli vs. disseminated GTD. The patient was transferred to a quaternary center, where she was started on broad-spectrum antibiotics and cisplatin/etoposide for two cycles prior to full-dose chemotherapy with etoposide, methotrexate, actinomycin D, cyclophosphamide, and vincristine due to concern of acute hemorrhage given her high tumor burden. Bacterial cultures of blood, urine, and tracheal aspirate were negative.

**Figure 3:**
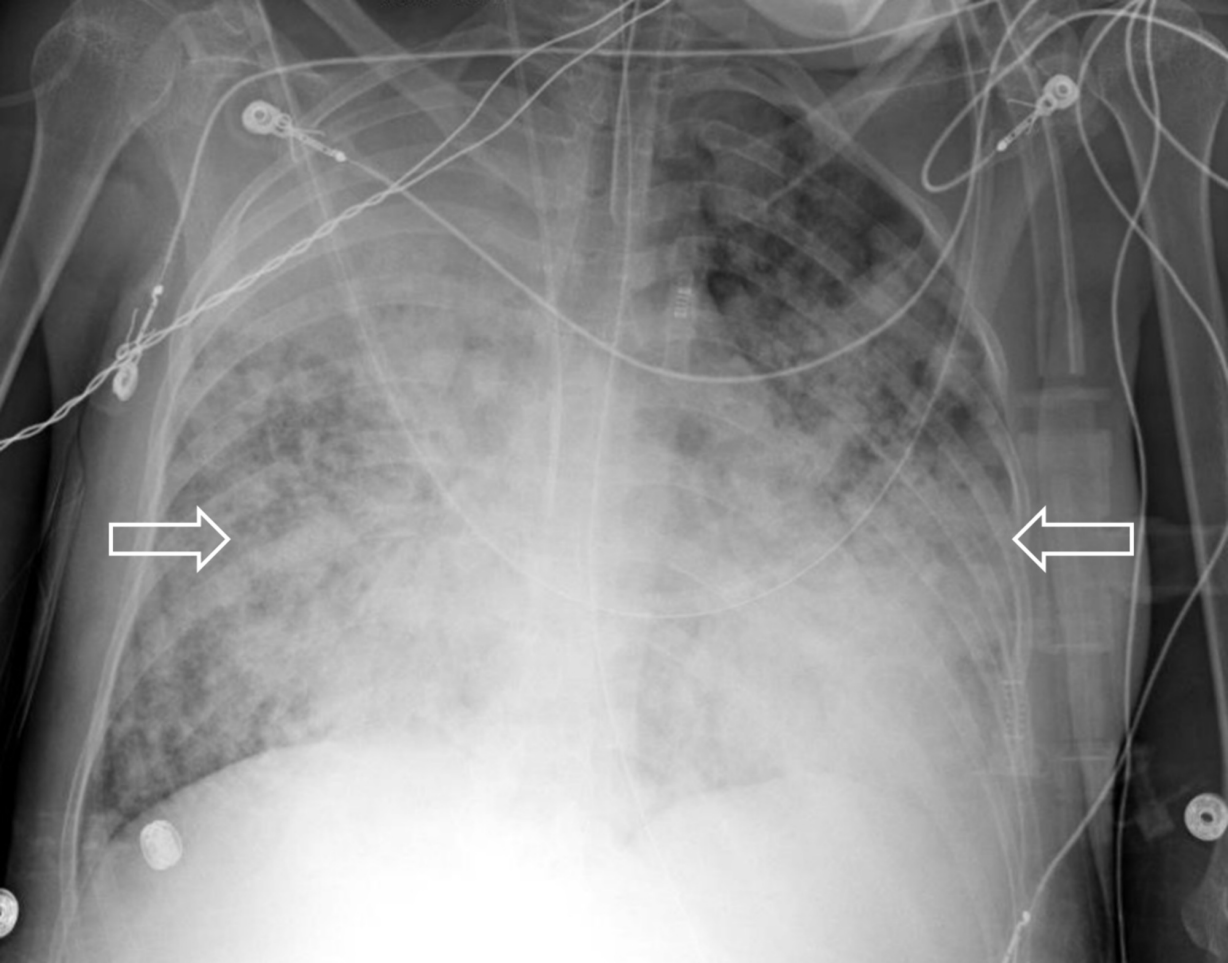
Chest X-ray on hospital day seven, revealing worsening ARDS and DAH (white arrows). ARDS, acute respiratory distress syndrome; DAH, diffuse alveolar hemorrhage

On hospital day seven, the patient developed massive hemoptysis with marked respiratory distress. Bronchoscopy showed active hemorrhage with predominant bleeding from the right upper lobe. The patient underwent interventional radiology-guided embolization of her right bronchial artery. She continued to have DAH (Figure 3).

Recruitment maneuvers (pressure-controlled inflations of 40 cm H2O sustained for 20 s) and neuromuscular blockade failed to improve gas exchange on assist-control ventilation with a TV of 4.5 mL/kg ideal body weight, RR of 23, a PEEP of 16 cm H2O, and an inspired oxygen fraction of 100% while minimizing driving pressure. She was placed on veno-venous ECMO on hospital day eight.

Under ultrasound guidance, a 23 French drainage cannula was placed percutaneously in the left femoral vein, and a 21 F return cannula was placed into the right internal jugular vein. Blood was drained from the left femoral vein into the ECMO circuit. ECMO flows were initiated at 4 L/min with a sweep gas flow of 2 L/min, titrated to keep oxygen saturation >88% and pCO2 within normal parameters. The ECMO for Protective Ventilation (EMPROVE) protocol, a lung-protective strategy utilized at our institution, was initiated on the ventilator. The protocol has been described in detail elsewhere, but in brief, it utilizes either pressure control or airway pressure release ventilation to maintain a peak pressure ≤ 30 or a plateau pressure ≤ 25. The respiratory rate is lowered to 5-8 breaths per minute, and moderate PEEP is used to keep the lungs expanded during low TV ventilation. The FiO2 is set at ≤ 50%. ECMO provided a gradual improvement in hypercapnia over the initial 45 min (PCO2 48-42, pH 7.48-7.45). Using the EMPROVE protocol her TVs declined from ~300 to ~200 mL, reducing peak airway pressures from 46 to 30 cmH2O. Her respiratory rate was maintained at 7 and her PEEP at 14 cm H2O.

**Figure 4:**
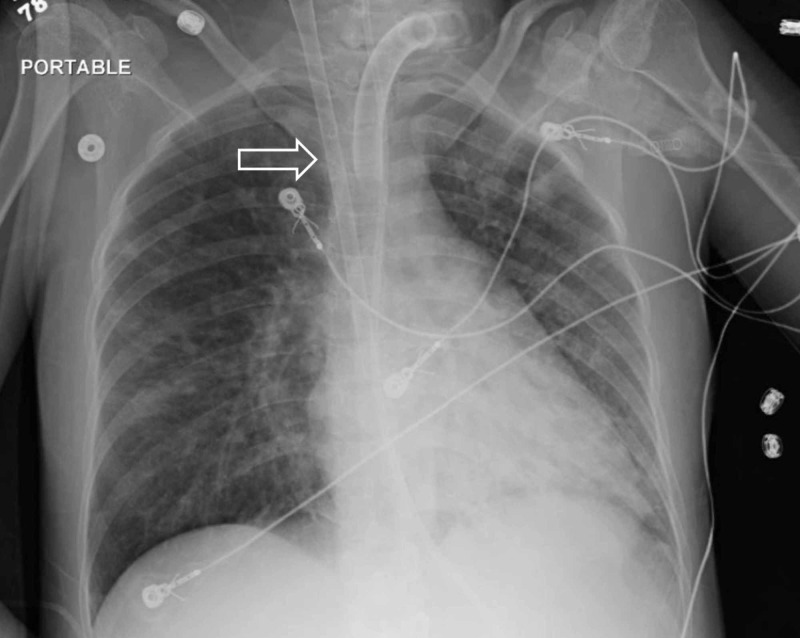
Chest X-ray on hospital day 31, showing resolved ARDS and ECMO cannula in situ (white arrow). ARDS, acute respiratory distress syndrome; ECMO, extracorporeal membrane oxygenation

Due to the hemorrhagic nature of GTD, the patient was not anticoagulated. Partial thromboplastin time measured between 25 and 36 s while on ECMO. Platelets were transfused to maintain count > 50,000/µL, as per local hospital practice. There were no thrombotic complications. The patient’s clinical condition gradually improved and extracorporeal support was discontinued after 23 days (Figure 4).

Sedation was weaned and she was alert and oriented, however, with significant debility. She underwent tracheostomy placement and was weaned to T-piece. Sadly, while nearing transfer status to a decreased level of care, the patient suffered a catastrophic hemorrhage from a brain metastasis and subsequently expired on hospital day 42.

## Discussion

Estimates for the incidence of various forms of GTD vary from one in 1500 pregnancies for hydatidiform moles to one in 20,000 to 40,000 pregnancies for gestational choriocarcinomas. Although patients with malignant GTD following nonmolar pregnancies may present with subtle signs and symptoms, approximately 20% of patients will develop malignant sequelae requiring administration of chemotherapy. Abnormal bleeding for six or more weeks following any pregnancy should be evaluated with hCG testing to exclude a new pregnancy or GTD. Metastases of GTD have been reported in virtually all body sites, most commonly the brain, liver, and lung. Standard treatment of GTD depends on clinical staging and risk categorization using the American Joint Committee on Cancer (AJCC) system. Suction and curettage under ultrasound control are essential, and low-risk GTD can be treated with a regimen of methotrexate with or without folinic acid and/or actinomycin-D. High-risk GTD is treated with methotrexate and actinomycin-D, with additional chemotherapeutic agents including etoposide, vincristine, cyclophosphamide, and/or cisplatin [1].

The pathophysiology predisposing to DAH in GTD stems from the unusually prominent vascularity of the underlying malignancy [2-3]. Acute respiratory failure should be anticipated in the setting of GTD metastatic to the lungs (AJCC stage ≥3), with high risk for the development of pulmonary hemorrhage. Treatment outcomes in such late-stage cases may depend highly upon survival through this critical stage [4]. Metastatic GTD involving the lungs with DAH requiring invasive mechanical ventilation with high PEEP is typically unresponsive to traditional rescue therapies [3, 5]. In severe circumstances, ECMO has been used as salvage therapy, maintaining acceptable gas exchanges, limiting ventilator-induced lung injury, and providing the necessary time to treat the underlying etiology. While there are case reports of ECMO use in patients with DAH and ARDS, experiences with ECMO in the post-partum period are limited, with no reported cases of ECMO use in patients with gynecologic malignancies.

DAH-related ARDS is associated with severe presentations and a high incidence of complications, especially in pregnant patients [4]. Use of ECMO in DAH has been reported in small case series with favorable outcomes [5,6]. Although no evidence-based literature exists to guide ECMO use in pregnant or post-partum patients with DAH, there is literature addressing ECMO use in such patients with influenza-related ARDS. ECMO in these instances has been reported in small case series; several of them during the 2009 pandemic of the swine-origin influenza A/H1N1, with favorable outcomes. One of the largest published experiences is from the national French registry, including all pregnant patients with laboratory-confirmed A/H1N1 influenza; Of the 315 patients included in the French registry, 40 were hospitalized in an ICU, and 11 required ECMO with a maternal survival rate of 82% [7]. Likewise, the largest single-center case series of pregnant or post-partum patients (N=18) showed a survival rate of 88% [8]. Furthermore, there are reports of successful ECMO treatment of ARDS secondary to less common etiologies, including staphylococcal septicemia, pulmonary hemorrhage, and varicella pneumonia. Although publication bias may exist, these studies support the utility of veno-venous ECMO as a rescue measure for patients with DAH and ARDS refractory to more conservative measures. Likewise, the CESAR study showed the effectiveness of ECMO in improving survival and quality-of-life in patients with severe ARDS, as the six-month survival in the ECMO cohort was 63% vs. 51% in the non-ECMO cohort [9].

In summary, despite patient demise due to metastatic disease and hemorrhage, our case demonstrates veno-venous ECMO is a feasible therapy in post-partum patients with severe ARDS secondary to DAH from disseminated GTD refractory to conventional rescue measures. The expanded use of ECMO in high-risk obstetric and gynecologic populations, such as those with ARDS and disseminated GTD, may result in improved maternal outcomes and warrants further investigation.

## Conclusions

In summary, despite patient demise due to metastatic disease and hemorrhage, our case demonstrates veno-venous ECMO is a feasible therapy in post-partum patients with severe ARDS secondary to DAH from disseminated GTD refractory to conventional rescue measures. The expanded use of ECMO in high-risk obstetric and gynecologic populations, such as those with ARDS and disseminated GTD, may result in improved maternal outcomes and warrants further investigation.
